# Clinical Correlates of Sports Betting: A Systematic Review

**DOI:** 10.1007/s10899-023-10196-0

**Published:** 2023-04-01

**Authors:** Eduardo Valenciano-Mendoza, Bernat Mora-Maltas, Gemma Mestre-Bach, Lucero Munguía, Jérémie Richard, Jeffrey L. Derevensky, Marc N. Potenza, Susana Jiménez-Murcia

**Affiliations:** 1 Clinical Psychology Unit, Bellvitge University Hospital, c/ Feixa Llarga s/n, 08907 Barcelona, Spain; 2 Universidad Internacional de La Rioja, La Rioja, Logroño, Spain; 3 Department of Educational and Counselling Psychology, McGill University, Montreal, Québec Canada; 4 Department of Psychiatry, Yale University School of Medicine, New Haven, CT USA; 5 Child Study Center, Yale University School of Medicine, New Haven, CT USA; 6 Connecticut Mental Health Center, New Haven, CT USA; 7 Connecticut Council on Problem Gambling, Wethersfield, CT USA; 8 Department of Neuroscience, Yale University, New Haven, CT USA; 9 Wu Tsai Institute, Yale University, New Haven, CT USA; 10 Department of Clinical Sciences, School of Medicine and Health Sciences, University of Barcelona, Barcelona, Spain; 11 Ciber Fisiopatología Obesidad y Nutrición (CIBERObn), Instituto de Salud Carlos III, Madrid, Spain; 12 Psychoneurobiology of Eating and Addictive Behaviors Group, Neurosciences Programme, Bellvitge Biomedical Research Institute (IDIBELL), Barcelona, Spain

**Keywords:** Gambling, Addictive behaviors, Impulsive behaviors, Compulsive behaviors, Sports betting, Psychopathology, Personality

## Abstract

Sports betting is becoming increasingly widespread, and a growing number of individuals, both adolescents and adults, participate in this type of gambling. The main aim of this systematic review was to assess correlates of sports betting (sociodemographic features, gambling-related variables, co-occurring psychopathologies, and personality tendencies) through a systematic review conducted following the PRISMA guidelines. Relevant studies were identified via searches of NCBI/PubMed and APA PsycInfo databases. Individuals from the general population and/or with a clinical diagnosis of gambling disorder (GD) were included, irrespective of gender and age. In addition, the studies needed to have administered at least one clinical interview/psychometric instrument to assess the presence of problematic gambling/GD, contain at least one group of participants with sports betting, and directly analyze the association between sports betting and any of the following features: sociodemographics, gambling-related variables, co-occurring psychopathologies, and/or personality tendencies. Fifty-four articles were included. Multiple sociodemographic variables have been studied in relation to sports betting. In general, males with high impulsivity have greater tendencies for sports betting. The co-occurrence of certain pathologies, especially substance use or other addictive disorders, was also suggested. Most studies were cross-sectional, assessed participants using self-administered instruments, recruited samples using non-probability online panels, included small samples, had unbalanced samples, and included samples from only one country. Impulsive males may be particularly prone to sports gambling and related problems. Future research should examine prevention strategies that may help prevent the development of sport-betting-related GD and other addictive behaviors in vulnerable individuals.

## Introduction

Over the last decade, the availability and accessibility of gambling has been expanding and evolving due to technological and sociological developments (Chóliz, 2016; Jiménez-Murcia et al., 2014). Consequently, consumers of gambling products have changed the way they behave and interact with these products, with a greater number of adults reporting gambling from home from their internet-connected devices (Lejoyeux, 2012). Online gambling has drastically increased the accessibility of gambling and as a result, the potential frequency of gambling and risk to experience symptoms of problem gambling (Hing et al., 2016; Lejoyeux, 2012). Of all the available forms of online gambling, sports betting is one of the most widely endorsed forms (Jiménez-Murcia et al., 2014; Mestre-Bach et al., 2022).

The popularity of online sports betting (OSB) has been continuing to increase in part due to its legalization in multiple jurisdictions globally (Lopez-Gonzalez & Griffiths, 2018). In 2018, sports betting was the most popular form of online gambling in Europe (European Gaming & Betting Association, 2019). With increasing legalization, sports betting establishments have proliferated, where other addictive substances such as alcohol could be consumed (Li et al., 2020). In recent years, novel features have been incorporated into sports-betting experiences, such as cash-out features, additional live in-play betting and request-a-bet options, instant deposits, and micro-betting (Lopez-Gonzalez et al., 2019; Winters & Derevensky, 2019). Nevertheless, the fact remains that sports gambling is often done through illegal bookmakers (Morgan Stanley, 2014) which has resulted in some researchers requesting legislators to develop measures for better regulation of the sports betting market (Hing et al., 2015a).

In addition to broadening availability, some studies have reported on the potential influences of advertising on the development of gambling problems as a result of online gambling including OSB (Bouguettaya et al., 2020; Newall et al., 2019). Despite the role of advertising on increasing the risk of gambling problems associated with OSB, individuals that develop problems with gambling tend to have certain predispositions that increase this risk (Chóliz, 2008; Hing et al., 2015a; Russell et al., 2019a, 2019b). Based on the Diagnostic and Statistical Manual Fifth Edition, gambling disorder (GD) is an addictive condition characterized by persistent and recurrent problematic gambling behavior that generates clinically significant levels of distress and impairments in functioning (American Psychiatric Association, 2013). Although the DSM-5 disorder does not specify types of gambling activities, there is evidence suggesting that certain forms of gambling may be associated with greater risks for the development of GD (Lutri et al., 2018; Stevens & Young, 2010; Williams et al., 2021). Due to sports betting in both online and offline forms increasing in popularity, it is essential to identify demographic and clinical characteristics associated with the risk for the development of GD associated with sports betting.

It is known that individuals engaging in OSB gambling represent a particularly vulnerable group, with a higher proportion of individuals who are single, younger, and of lower socioeconomic status, report an earlier onset of gambling participation, endorse higher rates of substance use disorders, report greater psychological distress and have distinct personality profiles (i.e., higher impulsivity, reward dependence, and novelty seeking) (Estévez et al., 2017; Granero et al., 2020). The evidence suggesting that OSB involves gambling participation among younger individuals is of particular concern as adolescents and emerging adults appear at increased risk of gambling problems (Sarabia et al., 2014).

Other relevant factors that may result in maintaining sports betting behaviors and increase the risk for problem gambling are cognitive biases (e.g., illusion of control). Cognitive biases may result in a reduction in the perception of risk and possible long-term effects as a result of gambling, and increase the perception that personal skills contribute significantly to gambling outcomes (Chóliz, 2010). However, cognitive biases specific to OSB remain understudied with inconsistent findings having been reported in their role for differentiating between problematic and non-problematic sports betting (Huberfeld et al., 2013).

Although there are increasing studies investigating the relationship between sports betting and problem/disordered gambling, many investigations conceptualize sports betting as one of the many forms of online gambling, rather than as an entity in and of itself. Moreover, few studies have examined the demographic and clinical characteristics of individuals reporting engagement in sports betting and whether certain characteristics differentiate between sports betting with or without problem/disordered gambling. The association between sports betting and GD has rarely been examined, particularly longitudinally, with no systematic review having been published on this topic. Despite the growing number of individuals experiencing clinically relevant problems with sports betting (Mestre-Bach et al., 2022), to the authors’ knowledge, no previous systematic review has been conducted examining the clinical characteristics of people engaging in sports betting. This marks a significant gap in the field that should be addressed as such information could facilitate the development of more effective prevention and treatment interventions (Winters & Derevensky, 2019). Taken together, the research question this systematic review aims to answer is: what are the clinical correlates (i.e., sociodemographic features, gambling-related variables, co-occurring psychopathology and personality tendencies) of people engaging in sports betting?

## Methods

### Study Selection

The methodology employed in this review adheres to principles of the Preferred Reporting Items for Systematic Reviews and Meta-Analyses (PRISMA; Moher, 2009). Relevant studies were identified via searches of NCBI/PubMed and APA PsycInfo databases using the following search terms: "sports bet*" OR "sports wager*" OR "sports gambl*" OR "fantasy sport*" OR "daily fantasy". The final search was conducted on May 28, 2021. No date range limits were applied to avoid selection bias. Only published or in-press empirical studies in peer-reviewed journals written in English, Spanish and French were considered for inclusion. In addition, only studies with an observational or descriptive design (e.g., cross-sectional, longitudinal, case–control) and a quantitative methodology were considered eligible for inclusion. Articles with no abstract, as well as publications that were not full articles, that had a qualitative design or that had the following specific formats (i.e., literature reviews, books, dissertations, case reports or series, editorials, clinical practice guidelines, commentaries, and gray literature) were excluded. Finally, studies evaluating treatment interventions were also excluded.

The present systematic review was performed on the basis of the following eligibility criteria: (1) human samples: general population (including both athletes and non-athletes) and/or individuals with a clinical diagnosis of GD, irrespective of sex and age; (2) administration of at least one clinical interview/psychometric instrument to assess the presence of problematic gambling/GD, (3) involvement of at least one group of participants with sports betting, and (4) direct analysis of associations between sports betting and any of these features: (A) sociodemographic characteristics (e.g., sex, age), (B) gambling-related variables (e.g., GD severity, gambling frequency, gambling-related cognitions, maximum bets), (C) co-occurring psychopathology, and/or (D) personality features (e.g., impulsivity).

Once duplicate results were excluded, all abstracts were screened for inclusion and exclusion criteria. Most studies excluded in this first step either investigated behaviors other than SB, had a dependent variable other than clinical correlates or were published in a language other than English or Spanish. For all studies identified for inclusion, a full text version was retrieved, and all studies were reviewed with regard to their quality and eligibility for the review. In case of exclusion of studies, reasons were documented. Included studies were read thoroughly, and the former defined measures were extracted and included in tables.

### Study Assessment and Data Extraction

A two-step process was performed to select the final articles included in the present systematic review. First, two reviewers (EVM and BMM) screened the titles and abstracts of all potential studies individually. For the second level of the screening, articles identified for full review were further screened according to the eligibility criteria by two separate authors (EVM and BMM). Differences in rating between both reviewers were resolved through consensus, with the assistance of a third reviewer (GMB). The complete screening process was conducted using Covidence, a software based on the PRISMA standard and recommended by Cochrane Reviews (Kellermeyer et al., 2018).

**Fig. 1:**
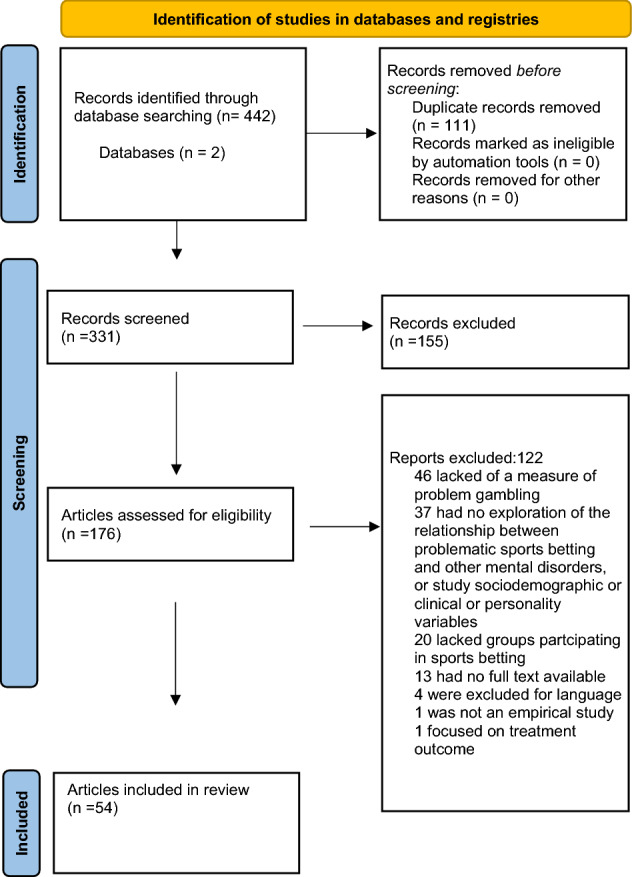
The PRISMA flow diagram of the selection process

A total of 442 records was retrieved from the literature search in the selected databases. After removing 111 duplicates, 155 of the 331 remaining articles were excluded taking title and abstract into account. The remaining 176 records were screened at a full-text level. From the 176 articles screened, 54 were ultimately included in the systematic review. Reasons for exclusion at the full-text screening stage are included in Fig. 1. Data were extracted including the full references of articles, main aims, study designs, sample characteristics and sizes, descriptions of methodology, and results.

### Risk of Bias Assessment

The risk bias of the included studies was performed using four items of the Effective Public Health Practice Project Quality Assessment Tool (EPHPP): selection bias, study design, data collection methods and appropriate statistical testing; additionally, a global rating was calculated (Thomas et al., 2004). The EPHPP is a guide to systematically appraise study quality in seven ambits: selection bias, study design, confounders, blinding, data collection methods, withdrawal and dropouts, and intervention integrity and analyses. The tool can be used to evaluate the study quality of observational, cross-sectional, before and after, and randomized controlled trial studies and has good content and construct validity and adequate test–retest reliability (Armijo-Olivo, Stiles, Hagen, Biondo, & Cummings, 2012; Thomas et al., 2004). Being that all the studies had cross-sectional designs, the items specific to intervention studies were excluded (i.e., confounders, blinding, withdrawals and dropouts, and intervention integrity). The study design criteria were also slightly modified to reflect the fact that most studies were cross-sectional, with differentiations based on sample size in both general population (Strong: *N* > 1250; Moderate: *N* ranging from 630 to 1249; Weak *N* < 630) and clinical samples (Strong: *N* > 100; Moderate: *N* ranging from 30 to 99; Weak *N* < 30) being included. Two authors (JR and LM) performed the quality assessment separately for each study, and discrepancies were resolved.

### Ethics

The present article is a systematic review of the literature, and no studies with human participants or animals were performed by any of the authors to conduct this work.

## Results

### Study Characteristics

Descriptions of the included studies and their findings are summarized in Table 1. Fifty-four articles were included in the systematic review. Australia was the country with the most published research on sports betting. All studies included in the systematic review had a cross-sectional design. Sample sizes ranged from 60 to 20,587. One study included only females (McCarthy et al., 2018), three studies included only males (Barrera-Algarín & Vázquez-Fernández, 2021; DiCicco-Bloom & Romer, 2012; Estévez et al., 2017), and the remaining 50 studies included both males and females. The percentage of males and females included in the last group of studies varied considerably, with the percentage of females being lower than that of males in most cases, with the exception of eight studies (Baggio et al., 2018; Hing & Haw, 2009).

**Table 1:** Description of the selected studies

1st Author, year	Aims	Design	Sample characteristics	Assessment					Results
				Sports betting	Problem gambling	Other gambling variables	Comorbidities	Clinical and other variables of interest	
(Baggio et al., 2018)	To compare associations of specific gambling activities with GD symptoms separately by gender	Cross-sectional (December 2013—May 2014)	Representative sample of people living in France N = 8805 Mage: females: 45.22 (SD = 0.22 ± years) males: 43.23 (SD = 0.23 ± years), Range 15–75 48.2% male; 51.8% female	Past-year engagement in sports betting	PGSI	Gambling preferences, gambling engagement	Substance use (past 12 months; alcohol, tobacco, cannabis, other illicit substances)	MH-5	Problem gambling was most strongly associated with gambling machines among females (most central variable to the network), whereas problem gambling was most strongly associated with sports betting, poker and casino gambling among males. Substance use in females had a weak relationship with sports betting, poker and casino gambling. For males, substance use had a larger number of associations with gambling, especially with sports betting, instant (scratch) lottery tickets, and poker
(Barrera-Algarín & Vázquez-Fernández, 2021)	To understand trends in gambling behaviors among individuals aged 18 to 30 years	Cross-sectional (March 2015—March 2019)	People in treatment with GD related to online gambling N = 188 Age: 85% between the age of 18 to 23; 15% between the age of 24 to 30, 100% male	Engagement in sports betting	DSM-5 diagnostic criteria SOGS	Other gambling activities, gambling-related cognitive distortions typical bet, money invested in gambling, means used to bet	AUDIT Cocaine Addiction Severity Test	BDI, HAM-A, CEPER, SCL-90, Self-esteem Scale Economic, social, personal, familial consequences of addiction, Rehabilitation process	Of the clinical sample, 78% reported engagement in sports betting, mostly using smartphones, tablets and mobile phones (97%) with the amount of money wagered ranging from $365 to $3651 at a time. Of the overall sample, 70% reported tobacco use disorder, 8% alcohol use disorder, 8% cocaine use disorder, 2% cannabis use disorder, 93% reported a debt greater that 5000 euros, problems with social support (8%), consequences at work and school (67–90%), high depressive symptoms (76%), high anxiety (90.6%), low self-esteem (96%), distrust (80%), communication problems (90–99%), and weak family cohesion (73%)
(Bodor et al., 2018)	To explore gambling activities and problem gambling among individuals with an alcohol use disorder in outpatient treatment	Cross-sectional	Alcohol dependent outpatients (based on ICD-10 criteria) currently in treatment N = 140 Mage = 53.09 (SD = ± 11.09 years) Range: 25–77 years	Past-year engagement in sports betting	SOGS	Gambling Activities Questionnaire (type and frequency)	Alcohol use disorder DSM-5 criteria		Sports betting was the third most commonly reported gambling activity (27.9%) yet was the gambling activity most frequently performed (12.2% gambled at least once a week, 3.6% daily, with other gambling activities ranging from 0–0.7% daily). The current sample of individuals with an alcohol use disorder often met criteria for being at-risk for problem/ pathological gambling (22.1%). Participants with a greater frequency of sports betting participation were more likely to be at-risk for problem gambling (*r* = .33), and pathological gambling (*r* = .37) compared to those with no problem-gambling symptoms. Overall SOGS scores were not correlated with self-reported DSM-5 symptoms of alcohol use disorder
(Brevers et al., 2021)	To investigate whether problem-gambling status, sports-betting passion and trait-level self-control modulate brain reactivity to sports-betting cues	Cross-sectional	People who frequently watch football matches with a history of sports betting on football N = 65 Mage = 26.04 (SD = ± 5.63 years) Range: 19–51 years 93.8% male; 7.2% female	Frequency of betting on football Sports-betting passion: Gambling Passion Scale	PGSI		Alcohol use disorder DSM-5 criteria	BSCS	Sport events not being available for betting elicited higher insular and striatal activation in people with versus without problematic sports betting. Lower trait-level self-control was associated with increased brain reactivity to sport events with high levels of winning confidence for matches that were not available for betting, suggesting that problem betting is linked to inaccessibility of gambling opportunities. No significant effect of sports-betting passion (harmonious or obsessive) was reported
(Cooper et al., 2021)	To examine differences in psychological characteristics and gambling behaviors between people with and without sports betting	Cross-sectional (2018)	People with past-year gambling N = 1280 Sports bettors: Male Mage = 44 years, Female Mage = 38 years. 54.3% male; 44.8% female Other bettors: Male Mage = 56 years, Female Mage = 48 years. 36.1% male; 63.7%% female	Past-year engagement in sports betting	PGSI	GMS, GBQ, Rational versus Experiential Scale, Gambling Identity Scale Type of gambling activity performed		UPPS-P	People who bet on sports versus those who did not were more likely to be male, younger, single, employed full-time, and college-educated; they also reported a higher income, gambling on a greater number of non-sport activities (3.29 vs. 1.60) and wagering a higher percentage of their income in a typical month (3.8% vs. 2.4%). People who bet on sports reported greater impulsivity scores with regards to negative urgency (Cohen d = 0.26), positive urgency (d = 0.34), and sensation-seeking (d = 0.52). People who bet on sports also reported a higher score on gambling identity items (eta = 0.24), greater scores on the gambling thinking style items (both experiential [d = 0.38] and rational thinking [d = 0.36]) and erroneous cognitions (both illusion of control [eta = 0.46] and luck/perseverance [eta = 0.40]) (all *p* < .001). People who bet on sports reported greater scores on each gambling motivation subscale including intellectual challenge (d = 1.02), excitement (d = 0.99), socialization (d = 0.83), monetary gain (d = 0.87), social recognitions (d = 0.68), and motivation (d = 0.49). People who bet on sports appeared at greater risk of problem gambling (eta = 0.29) which may be associated with differences in the number of gambling behaviors performed, attitudes towards gambling, impulsivity, thinking styles, erroneous cognitions, and gambling motivations (all *p* < .001)
(DiCicco-Bloom & Romer, 2012)	To examine the association between social status, gambling perceptions, poker playing, problem gambling and sports betting among adolescents	Telephone interviews (2002—2008)	High-school-aged youth population N = 2188 Age range: 14–18 years 100% male	Engagement in sports betting in an average week and month	DSM-IV for GD based on the NASY (4 symptoms; preoccupation, tolerance, withdrawal, loss of control)	Engagement in different types of gambling activities Perceptions of popular people and their gambling Number of friends who gamble		BSSS Engagement in clubs, organized or other extracurricular activities	In the model statistically predicting sports betting, non-Hispanic Black, friend's gambling, friend's approval of gambling, sensation-seeking, lottery gambling, slot-machine gambling, and participation in sports were significant statistical predictors. With regards to the four symptoms of problem gambling and sports betting, preoccupation and tolerance were statistically predictive of sports betting, whereas subjective loss of control and withdrawal were not
(Dwyer et al., 2018)	To examine problem-gambling severity in conjunction with participation in DFS, motives, perceptions and consumer behavior	Cross-sectional	Individuals reporting engagement in DFS (or reporting season-long participation) N = 546 Mean age range from 31.3–39.2 years Male sex range from 88–97% White ethnicity range from 76 to 83% of each group (based on problem-gambling severity)	Engagement in DFS, hours watching NFL games. Number of fantasy lineups entered per week, spending, average won/lost per week	PGSI	MSSFP. Sport attachment factor from the Points of Attachment Index			Among individuals engaged in DFS, a total of 144 participants were classified as having non-problem gambling, 194 with low-risk gambling, 133 with moderate-risk gambling, and 75 with high-risk gambling. The primary factor differentiating these groups was the mean score on financial motives, although motives for social interaction and competition were also significant. People with problem gambling also endorsed a greater number of lineups per week. In the regression model, perceived competitiveness (B = .28), financial motives (B = .26), self-reported amount of money won per week (B = .21) and self-reported number of lineups per week (B = .14) statistically predicted problem gambling. Entertainment/escape was a significant negative statistical predictor of problem gambling (B = -.16)
(Estévez et al., 2017)	To determine the differences between adults with GD who exclusively bet on sports online, patients with non-sports internet gambling and patients with offline gambling	Cross-sectional (2005—2015)	Males seeking treatment for problem gambling N = 2743 Mage = 42.19 (SD = ± 13.44 years) 100% male Ethnicity: 92.9% Spanish, 7.1% Immigrant	Past-year engagement in sports betting	DSM-5 criteria for GD SOGS	Type of gambling activity. Age of onset Mean and maximum monetary spending in a single gambling episode Debts Gambling activity preferences	Substance use and abuse (tobacco, alcohol, other drug)	TCI-R	People who gambling online and not on sports were more likely to be single, be younger, have higher monthly incomes, have begun gambling earlier and have the shortest durations of GD compared to people who bet on sports and gambled offline. People who bet on sports online made higher maximum bets. The online groups had higher cumulative debts compared to the offline group. People who bet on sports online obtained higher scores in persistence (industriousness, determination, ambition, perfectionism) compared to people who bet on online but not on sports and people who bet offline. As for substance use and abuse, no statistically significant differences were identified
(Gainsbury et al., 2019)	To examine the relationships between specific gambling activities and modalities (internet and venue-/land-based) to GD and general psychological distress	Cross-sectional (March 2017—April 2017)	People with past-month internet gambling N = 998 Mage = 48.18 (SD = ± 15.81 years), Range: 18–85 years. 57% male; 43% female Ethnicity: 82% European, 7% East/Southeast Asian, 5% South Asian, 2% Middle Eastern, 2% Indigenous Australian, 2% Other	Engagement in sports betting over the past month	PGSI	Online and land-based gambling frequency Attitudes towards gambling sites. Motivation for online gambling		Psychological distress (K6)	Overall, 50.1% of participants gambling online reported past-month sports betting (32% reported weekly or daily), this percentage being 35.57% of people with venue-based gambling (20% report weekly or daily). Online and venue-based sports betting were correlated at *r* = 0.66 (*p* < .001). Problem gambling was correlated with a greater frequency of sports betting online (*r* = 0.31, *p* < .001), and sports betting in venue (*r* = 0.43, *p* < .001). When controlling for overall gambling frequency, problem gambling was significantly positively associated with the frequency of online and venue-based gambling using electronic gaming machines (EGMs) and venue-based sports betting (B = 0.13, SE = 0.06, *p* = .039). Psychological harms were correlated with frequency of sports betting online (*r* = 0.18, *p* < .001), and sports betting in venue (*r* = 0.30, *p* < .001). Psychological distress was uniquely associated with higher frequency of venue gambling using EGMs, sports betting (B = 0.15, SE = 0.06, *p* = .011), and casino card/table games
(Grall-Bronnec et al., 2016)	To estimate the prevalence of gambling (problem or not) among European professional athletes and explore factors associated with gambling and problem gambling	Cross-sectional (July 2013—March 2014)	European professional athletes (PAs) N = 1,236 Mage = 25.2 (SD = ± 5.1 years), Range: 15–52 years 98.3% male; 1.7% female Ethnicity: 89.3% European (33.7% from Sweden)	Frequency of sports betting	Lie/Bet questionnaire based on two DSM-IV criteria for GD	First gambling, frequency, favorite type of gambling, preferred way of gambling, betting on one's own sport or own team Gambling-related cognitions		UPPS-Short Form	The most commonly reported gambling activity was sports betting (55.5% of the 882 PAs reported lifetime gambling), with 37% reporting betting on their own sports and 4% betting on their own team. In predicting problem gambling, betting on one’s own team (OR = 4.1), betting online (OR = 2.9), gambling regularly (OR = 4.0) and having a high positive urgency score (OR = 1.5) were associated with current or past gambling problems
(Granero et al., 2020)	To explore the existence of latent classes associated with gambling behaviors/ tendencies among people seeking treatment for online sports betting	Cross-sectional (2005—2019)	Online sports betting N = 323 Mage = 32.2 (SD = ± 9.7 years), Range: 15–80 years 96% male; 4% female	Engagement in online sports betting	DSM-IV-TR and DSM-5 criteria	Age of onset, duration, bets per gambling/ episode, cumulative debts	Substance use	SCL-90-R TCI-R	The prevalence of people seeking treatment for online sport betting appears to be an increasingly common reason for consultation from 2005 (0.3%) to 2019 (16.1%). Of people seeking treatment for online sport betting, 49.8% reported substance use and 71.2% a secondary behavioral addiction. Two latent clusters were identified. Cluster 1 (*n* = 247, 76.5%) grouped patients who were more severly affected by online-sport-betting behaviors. These patients were characterized by being non-married, having lower socioeconomic level, using substances (alcohol, tobacco and other), being of younger age, having earlier onset of gambling, experiencing higher debts due to online sport betting, having higher psychopathological distress (based on overall SCL-90R scores), and demonstrating more dysfunctional personality profiles (higher novelty seeking, lower levels of self-directedness and cooperativeness). Cluster 2 (*n* = 76, 23.5%) grouped patients who were less affected by online-sport-betting behaviors. These patients were mostly married (or living with a stable partner), with higher socioeconomic levels, older age and later onset of the gambling activity, less substance use, and more functional psychopathological and personality profiles
(Håkansson, 2020)	To understand online gambling behaviors during the COVID-19 pandemic, including online sports betting	Cross-sectional (May 2020)	People with past-year online gambling N = 997 Age range: 18–69 years 75% male; 25% female	Past-year and past-month engagement in sports betting	PGSI	Past-year and past-month participation in different types of gambling			In the total sample, 23% had low-risk gambling, 15% moderate-risk, 10% problem gambling, with a greater percentage of women reporting problem gambling (15% vs 6%). Having moderate-risk or problem gambling was more likely among people who bet on sports. Comparing people who bet on sports in the past month versus to past year, the former group was more likely to report other forms of gambling. The group was also more likely to report past indebtedness and greater severity of gambling problems (moderate-risk and problem gambling of 18% and 13%, respectively)
(Håkansson et al., 2017)	To understand the characteristics of patients seeking treatment for disordered gambling, including associations with gambling type, psychiatric comorbidity and gender	Retrospective patient chart review in treatment center (January 2016—April 2017)	Individuals seeking treatment for problem gambling at an outpatient facility N = 106 Mage = 31.5 (SD = NR), Range: 18–72 years 80% male; 20% female	Past-year engagement	DSM-5 diagnostic criteria	Problematic types of gambling participation and main types including sports betting	Mood disorders, substance use disorders		Of those seeking treatment, 58% received a psychiatric disorder apart from GD (including substance use, mood, anxiety, stress-related, somatoform and attentional disorders). Problematic gambling on online casino or online sports betting represented 84% of patients, with 41% reporting online sports betting. Online sport betting was associated with gender, with 53% of males reporting this compared to 0% of females
(Håkansson & Widinghoff, 2020)	To examine whether specific gambling patterns are related to problem gambling and over-indebtedness, controlling for psychological distress, gender and other potential risk factors	Cross-sectional	People with online gambling from the adult general population N = 1004 Age range: 18–70 + years 78% male; 22% female	Sports betting involvement over the past 30 days	PGSI	Gambling participation and day gambling losses History of subjective indebtedness Expected over-indebtedness	Substance use (including tobacco)	K6 Felt need to seek treatment for alcohol or drug problems	Overall, 54% of the sample reported live sports betting and 60% reported non-live sports betting. In the total sample, 23% had low-risk gambling, 19% had moderate risk, and 13% had problem gambling. Moreover, 4% of those reporting only live sports betting had problem gambling, and an additional 21% were in the moderate-risk range. A total of 12% of the sample reported a history of over-indebtedness and 8% expected over-indebtedness, this being reported by only 4% of people who bet on live sports. Problem gambling was associated with psychological distress, recent online casino gambling, and recent combined online casino gambling and live sports betting (OR = 5.12), but not live-sports betting alone. History of over-indebtedness was associated with recent combined online casino gambling and live sports betting (OR = 1.57), but not live-sports betting alone
(Hing et al., 2019)	To assess whether perceived attractiveness of gambling activities varies based on the amount and type of information provided about their play-through conditions, individuals’ comprehensions of their true costs, and whether individuals’ comprehensions of their true costs varies based on problem-gambling severity	Cross-sectional (May 2017—June 2017)	People who bet on sports N = 299 Age range: 18–39 years 96% male; 4% female	Past-year engagement in sports betting	PGSI				In a sample of people who bet on sports, overall, 27.4% had low-risk gambling, 38.12% moderate-risk gambling and 18.7% problem gambling. There were an absence of group differences in the ratings of attractiveness of advertising, and the estimated winnings as calculated based on the advertising. Overall, participants tended to underestimate the median bet requirement necessary to withdraw winnings
(Hing & Haw, 2009)	To develop a scale measuring gambling accessibility	Cross-sectional	Gambling venue employees in clubs and hotels who reported gambling N = 533 Median age = 40 years, Range: 18–70 years 32.8% male; 67.2% female	Past engagement in sports betting	PGSI	Gambling accessibility based on social, physical and cognitive accessibility			The physical accessibility subscale was correlated with the PGSI scores of people who bet on sports (*r* = 0.24, *p* < .05). The cognitive accessibility subscale was correlated with sports-betting expenditure (*r* = 0.22, *p* < .05) and sports-betting frequency (*r* = 0.23, *p* < .05)
(Hing et al., 2018a)	To identify behavioral, psychological and socio-demographic statistical predictors of impulsive sports betting	Cross-sectional	People who bet on sports N = 1816 Age range: 18–60 + years 69% male; 31% female	Past-year engagement, age of first bet, number of accounts, expenditure, days wagering on sports in an average month, number of bets in a day, methods used, type of sports betting	PGSI	Impulsive betting		BIS-Brief	Overall, 16.2% of the sample had low-risk gambling, 17.6% moderate-risk gambling and 46.8% problem gambling. Impulse betting was common, accounting for 46.6% of all past-year sports bets by respondents. 78.4% of respondents had placed one or more impulse bets in the prior year and 15.2% of respondents had made all of their sports bets on impulse. People who bet on sports who placed wagers before the match were characterized by higher trait impulsiveness (B = 0.20, *p* < .001), male sex (B = 0.14, *p* < .001) and a lower percentage of sports bets on the final match outcome (B = -0.09, *p* = .018). More impulsive people who bet on sports during the match were characterized as having: higher problem-gambling severity (B = 0.051, *p* = 0.036), more frequent sports betting (B = 0.055, *p* = 0.003) and a shorter history of sports betting (B = 0.041, *p* = 0.029), with fewer bets placed over the internet (B = -0.071, *p* < .001) and more wagers on micro-bets/in-game contingencies (B = 0.218, *p* < .001)
(Hing et al., 2017)	To determine potential demographic, behavioral and psychological risk factors for problem gambling for individuals gambling betting on online EGMs, online sports and online races and to compare the characteristics of groups with problem gambling on each of these online forms	Cross-sectional	People with past-year gambling N = 4594 People with non-problematic online sports betting: Mage = 41.3 (SD = ± 14 years). 90.4% male; 9.6% female. Ethnicity: 84.4% Australian People with problematic online sports betting: Mage = 31.1 (SD = ± 9.8 years). 98.3% male; 1.7% female. Ethnicity: 76.2% Australian	Past-year involvement in online sports betting	PGSI	Frequency of engagement in 10 forms of gambling over the prior 12 months Most problematic modes and forms of gambling, gambling attitudes	Substance use: alcohol or illicit drug use when gambling	K6 Help-seeking in relation to gambling problems	Comparing groups with non-problematic online sports betting to problematic online sports betting, individuals with problematic online sports betting were more likely to be gamble on sports offline and were more likely to consider themselves semi-professional gamblers. They were also more likely to use illicit drugs while gambling and reported greater psychological distress. Potential risk factors for online sports betting were being male (B = 1.35, *p* = .032), younger (B = -0.07, *p* < .001), having been born outside of Australia (B = -0.66, *p* = .017), speaking a language other than English (B = -0.66, *p* = .017), engaging in more frequent sports betting (B = 0.65, *p* < .001), and having more negative attitudes toward gambling (B = -0.33, *p* < .001). Compared to people with problematic EGM gambling, those with problematic online sports betting were younger, wer more educated, and engaged in fewer forms of gambling. Compared to people with problematic online race betting, those with problematic online sports betting were younger and less likely to have been born in Australia
(Hing et al., 2016)	To compare gambling behaviors, problem gambling symptoms, related harms, recognition and help-seeking among people with problem semi/professional gambling and problem amateur gambling	Cross-sectional	People with amateur and semi/professional gambling N = 4594 Problem (n = 57), semi/professional gambling(n = 311). Mage = 32.7 (SD = ± 11.5 years). 88.7% male; 11.3% female Problem amateur gambling (n = 4226), Mage = 38.5 (SD = ± 13.1 years). 78.9% male; 21.1% female	Past-year involvement in online sports betting	PGSI	Self-perception as professional, semi-professional or amateur/recreational Frequency of engagement in 10 forms of gambling Most problematic modes and forms of gambling Gambling-related harms		Help-seeking behaviors	People with problem semi/professional gambling were more likely to have problems with sports betting compared to those with problem amateur gambling
(Hing et al., 2016)	To identify potential demographic, behavioral and normative risk factors for gambling problems among people who bet on sports	Cross-sectional (October 2012—December 2012)	People who bet on sports N = 639 63.9% male; 36.1% female	Past-year participation and expenditure Number of agencies where accounts were held Timing of sports bets, impulse betting, percentage of sports bets placed before and during match	PGSI				People who bet on sports appearing at a higher risk of problem gambling were those who were young, male, single, educated, and employed full-time (or were full-time students). People who bet on sports appearing at risk of problem gambling were found to have greater frequency and expenditure on sports betting, greater diversity of gambling involvement and more impulsive responses to betting opportunities, including in-play live action betting (as opposed to betting before a game). Normative influences (i.e., higher subjective norms) from media advertising and from significant others were also associated with greater problem-gambling risk
(Hing et al., 2017)	To examine whether responses to sports-betting promotions vary by problem-gambling severity	Cross-sectional (October 2012—December 2012)	People who bet on sports online N = 455 Age = 39.4% aged 18–34; 37.6% aged 35–54; 23.2% aged 55 + 71.5% male; 28.5% female	Past-year participation	PGSI	Attitudes towards promotion of gambling during televised sport Approval of gambling promotional techniques Subjective influence of gambling promotions on sports betting behaviors			Young males who bet on sports appeared especially vulnerable to gambling problems (*r* = -0.35, *p* < .001), particularly if they held positive attitudes to gambling sponsors who embedded promotions into sports broadcasts (*r* = -0.27, *p* < .001). In the regression model, PGSI scores were statistically predicted by: being male (B = 0.39, *p* = .007), younger (B = -0.26, *p* < .001), having more favorable sponsorship responses (B = 0.001, *p* = .032), having higher approval of gambling promotional techniques (B = 0.23, *p* = .043), and demonstrating greater influences of gambling promotions on sports-betting behaviors (B = 0.76, *p* < .001)
(Hing et al., 2018b)	To examine whether the uptake of wagering inducements statistically predict impulse sports betting	Cross-sectional (July 2016—September 2016)	People who bet on sports N = 1813 Mage = 35.3 (SD = ± 12.7 years) 68.9% male; 31.1% female	Past-year participation	PGSI	Types of bets: researched or planned, impulse before the start of a match, impulse during the match		Buying Impulsiveness Scale	People who bet on sports with lower overall problem-gambling symptoms were more likely to place bets that were researched and planned in advance of the match. More frequent use of wagering inducements was related to placing impulsive in-play bets. Impulse in-play bets were also statistically predicted by problem gambling (B = 0.16, *p* < 0.001), higher buying impulsiveness (B = 0.06, *p* = 0.015), higher frequency of watching sports, younger age, and higher educational status. People who bet on sports with greater tendencies to place impulse bets before commencement of matches also tended to have higher buying impulsiveness (B = 0.22, p < .001) and were younger, but they reported using inducements less frequently, were more likely to be female, were less educated and reported fewer symptoms of problem gambling
(Hing et al., 2015a)	To explore whether exposure and attitude to gambling promotions during televised sport statistically predicts sports-betting intention and whether this relationship varies with problem-gambling severity	Cross-sectional (October 2012)	Adults N = 1000 Age range: 18–85 years 49.5% male; 50.5% female	Past-year participation Attitude to the promotion of gambling and to promotional techniques during televised sports Perceived behavioral control with sports betting	PGSI	Gambling participation over the past year			Overall, 78.2% of participants had non-problem gambling, 9.7% had low-risk gambling, 6.2% had moderate-risk gambling, and 5.9% had problem gambling. Strongest statistical predictors of greater intended frequency of sports betting in the next six months were greater problem-gambling severity (B = .45, *p* < .001), previous sports-betting participation (B = .20, *p* < .001), more frequent exposure to the promotions (B = .11, *p* < .01), and more positive attitudes towards them (B = .09, *p* < .05)
(Hing et al., 2015b)	To explore among people who bet on sports their responses to gambling promotions and whether these vary with problem-gambling severity	Cross-sectional (October 2012)	People who bet on sports N = 544 Mage = 42.2 (SD = ± 14.26 years), Range: 18–80 years 63.6% male; 36.4% female	Past-year participation Proportion of sports bets according to the use of venues Proportion of bets planned and on impulse	PGSI	Frequency of gambling on seven main types of gambling Subjective impact of gambling promotions			People who bet on sports frequently participated in other gambling activities. Among the 544 people who bet on sports, 273 (50.2%) were classified as having non-problem gambling, 97 (17.8%) as having low-risk gambling, 54 (9.9%) as having moderate-risk gambling, and 120 (22.1%) as haivng problem gambling. Compared to those with non-problem and at-risk gambling, people with problem gambling reported the most encouragement and influence to gamble from gambling promotions. Specific impacts included increasing the frequency of sports betting, expenditures on sports betting, increased time spent on sports betting, spending more money on sports betting than intended, spending more time on sports betting than intended, and experiencing greater sports-betting-related harms
(Holtgraves, 2009)	To examine similarities and differences between gambling activities, gambling frequency and rates of problem gambling	Cross-sectional (2001—2005)	Adults N = 12,299	Past-year participation	PGSI	Frequency of gambling in eight activities over the past year			The factor structure suggests that certain people prefer different gambling activities, with one group preferring to gamble on lotteries, bingo, EGMs, and raffles, and a second group preferring to gamble on the internet, horse races, and sports
(Jiménez-Murcia et al., 2021)	To explore determinants of sports-betting severity in Spain based on psychopathological distress and personality factors	Cross-sectional (2005—2020)	Spanish adults seeking treatment for problems related to sports betting N = 352 Mage = 32.1 (SD = ± 9.62 years), Range: 14–70 years 96.3% male; 3.7% female	Past engagement	DSM-IV-TR and DSM-5 criteria SOGS			SCL-90R TCI-R	Among Spanish people who bet on sports, older age (B = 0.006, *p* = .02), higher psychopathological distress (B = 0.14, *p* < .001), lower self-directedness (B = -0.003, *p* = 0.048), and higher novelty seeking (B = 0.005, *p* < .001) were statistical predictors of problem-gambling severity. The highest betting frequency was reported in men (B = 0.93, *p* = 0.007), with the lowest education levels (B = 0.31, *p* = .003), but higher social status (B = -0.35, *p* = .001), higher psychopathological distress (B = 0.41, *p* = .001), reward dependence (B = 0.01, *p* = .013), self-transcendence (B = 0.02, *p* = .017) and lower persistence (B = -0.01, *p* = .007) were also identified. Gambling-related debts were associated with a higher score in cooperativeness (B = 0.03, *p* = .004), male sex (B = 1.31, *p* = .045), unemployment status (B = -0.60, *p* = .03), psychopathological distress (B = 0.50, *p* = .048), novelty-seeking (B = 0.02, *p* = .032), and cooperativeness (B = 0.03, *p* = .004)
(Li et al., 2020)	To explore how food or substance consumption (e.g., experiencing hunger, or having consumed alcohol or recreational drugs) could shape consumer impulsive spending on sports-betting products	Cross-sectional	People who bet on sports online N = 1211 Age groups: 18–24 (19.6%), 25–34 (39.1%), 35–44 (24.4%), 45–54 (9.2%), 55–64 (5%), 65 or older (2.7%) 65.6% male; 34.4% female	Impulsive betting behavior (bet timing and bet size); promotional, social and financials influences	PGSI			Hunger; alcohol consumption; recreational drug consumption	Among people who bet on sports online, as participants’ hunger level, alcohol consumption, recreational drug consumption, or PGSI scores increased, their impulsive bet size increased as well. Participants who were hungrier when placing an impulsive bet, or had consumed more alcohol or more recreational drugs prior to placing the bet, tended to spend more on the bet. Participants who were hungrier, or had consumed more alcohol or more recreational drugs, tended to be more susceptible to promotional and financial influences, which were subsequently related to spending more on an impulsive bet
(Li et al., 2015)	To develop a cluster typology of people who bet on sports and to describe the clusters according to demographic and behavioral characteristics	Cross-sectional	Residents in mainland China who purchased sports lottery tickets more than once in the 12 months prior to the study N = 4,980 Age groups: ≥ 20 years (2.9%), 21–30 (29.7%), 31–40 (26.6%), 41–50 (21.2%), 51–60 (14%) and ≥ 61 years (5.6%) 77.3% male; 22.7% female	Expenditure, frequency of sports-lottery purchasing in a week, time commitment to daily sports-lottery-related activities, and types of sports lotteries purchased	SAPG				In the general population that purchased sports-lottery tickets, four clusters were identified. Cluster I, people who gambled casually (45%), had the lowest score on each dimensions of the SAPG, with no negative impact from gambling or any psychological symptoms from problem gambling. Cluster II, people who gambled in an escalating fashion (28%), had higher scores on all dimensions of the SAPG than those in Cluster I, especially on the compulsive disorder dimension. This cluster was considered a group whose gambling might escalate in the future. Cluster III, people who gambled in an at-risk fashion (11%), had significant social and financial problems that may need social intervention to address and resolve concerns associated with gambling. Cluster IV, people who gambled in a compulsive fashion (10%), had higher scores on the compulsive disorder and over expectation dimensions of the SAPG. These players may need psychosocial intervention to address and resolve concerns associated with gambling
(Lopez-Gonzalez et al., 2018)	To empirically validate the adaptation of the PGSI to Spanish-speaking countries	Cross-sectional (2017)	People who bet on sports N = 659 Mage = 35.1 (SD = 10.12 years) 74.2% males, 25,8% females	Sports-betting involvement	PGSI				Among people who bet on sports, the non-problem gambling group comprised 38.8% of the sample, low-risk gambling 26.6%, moderate-risk gambling 15.5%, and problem gambling 19.1%. Of the low-risk gambling group, 64.5% reported chasing their losses, whereas 17.7% felt guilty about their gambling, and 13.1% felt criticized for their gambling. People with problem gambling were more likely to live only with their partner
(Lopez-Gonzalez et al., 2020)	To compare Australian and Spanish people who gamble to identify similarities based on socio-demographic profiles, online or offline betting activity, and devices used to bet, while assessing in-play betting in both countries	Cross-sectional (2016 in Australia; 2017 in Spain)	People who bet on sports N = 1099 Mage = 35.70 (SD = 12,25 years) 79.3% males; 20.7% females Australian (n = 738) and Spanish (n = 361)	Channel used to bet, device used, and engagement with in-play betting, and the in-play betting behavior	PGSI				PGSI scores were significantly higher in the Australian sample, whereas Spanish people who bet on sports were more likely to have non-problem or low-risk gambling. In the Australian sample, being classified as having problem gambling was associated with being younger, female, having undertaken higher education (vs. high school or less), preferring to bet offline or via other channels (vs. online), preferring to bet via a combination of two or more devices (vs. desktop/laptop), and placing a higher proportion of in-play sports bets. In the Spanish sample, being classified as having problem gambling was associated with preferring to bet offline, preferring to bet via tablet, and placing a higher proportion of in-play sports bets. Significant interactions were observed for age and proportion of in-play sports bets, with all relationships being significantly stronger in the Australian sample
(Lopez-Gonzalez et al., 2019)	To explore the association between the use of new structural characteristics of online betting and problem-gambling severity	Cross-sectional (2017)	People who had bet on sports over the prior year N = 659 Mage = 35.1 (SD = 10.1 years) 74.2% males; 25.8% females	Live in-play betting; cash-out feature use; fantasy sports; location of betting; and device or platform used to make bets	PGSI				Problem-gambling severity was positively associated with how often participants discussed betting with other people before placing bets, how often they used new online betting functionalities and devoting more time to betting. In-play betting was more prevalent among people with problem gambling compared to any other group. Overall, 94.4% of people who bet on sports in the problem-gambling category had engaged in fantasy sports. A significant correlation was identified between scores on fantasy sports participation and severity of problem gambling. Most people who bet on sports across every problem-gambling severity category (75.11%) bet from home. Online gambling was more prevalent than offline gambling. Online betting was reported by 90.47% of people with problem gambling as their preferred way to gamble
(Lopez-Gonzalez & Griffiths, 2021)	To examine whether problem gambling is associated with gambling advertising impact in three dimensions including overall influence that people who bet on sports think gambling advertising have on their behavior, how knowledgeable people who bet on sports are about sports betting brands, and how similar people who bet on sports feel they are in relation to the main characters that are featured in sports-betting advertisements	Cross-sectional (2017)	Adults who had bet on sports over the prior year N = 659 Mage = 35.1 (SD = 10.1 years) 74.2% males; 25.8% females	Impact of Sports Gambling Promotions on Behavior Knowledge of sports-betting brands Similarity to Story Character adapted to sports-betting brands	PGSI				Among those individuals who bet on sports, no differences were identified based on age or gender in terms of advertising impact on the individual. People experiencing more severe gambling problems also reported more knowledge of bookmakers’ brands, more similarity to the main story characters in sports-betting advertisements and a higher perceived influence of advertising on their behavior
(Marchica et al., 2017)	To investigate how frequency of sports betting, DFS and league-based fantasy sports participation relates to disordered gambling behaviors among adolescents and to understand the frequency and severity of sports-related gambling, and the gender- and age-related differences in sports wagering	Cross-sectional (2016)	Junior-high and high-school adolescents N = 6818 Mage = 14.9 (SD = 1.76 years). Three age groups 10–12 years (N = 669); 13–15 years (N = 3219); and 16–19 years (N = 2675) 49% male; 51% female White (79%)	Frequency of gambling, seasonal fantasy sports betting, DFS betting, and sports betting in general	DSM-IV criteria NODS-CLiP				Regular involvement in sports betting, fantasy sports betting, and DFS betting among adolescents was associated with greater likelihoods of gambling problems. Males participated more frequently in these activities, while females who reported sports betting had a greater likelihood of being at-risk for problem gambling. Students aged 16–19 years old were at a higher risk for developing a gambling problem compared to younger adolescents when regularly engaging in sports-related gambling. Regularly participating in DFS was the strongest statistical predictor of at-risk gambling among 13–15 year-old students. All forms of sport-relevant gambling activities were significant statistical predictors of at-risk gambling
(Marchica & Derevensky, 2016)	To assess rates of participation in fantasy sports among National Collegiate Athletic Association (NCAA) student-athletes over an eight-year span, while considering differences in gender-related participation and to assess the potential risk of at-risk/probable-problem gambling among people who gamble on fantasy sports	Three cross-sectional studies (2004, 2008 and 2012)	Student-athletes N = 2004: 19,354 athletes; 2008: 19,371 athletes; 2012: 22,935 athletes Male: 2004 = 70.7%; 2008 = 66%; 2012 = 57% Female: 2004 = 48.9%; 2008 = 39%; 2012 = 39%	Past-year engagement	DSM–IV–TR	GAQ			An increase in fantasy sports participation was observed over time (15.3% from 2004 to 2008; 0.7% increase from 2008 to 2012). Of the total sample, 20% reported participating in fantasy sports games for a fee in 2012. In 2008, 71.9% and in 2012, 37.6% reported they considered participating in a fantasy league with fees and prize money as gambling. Male participation in free fantasy sports leagues was 6–7 times more likely compared to females. Among male students at risk for or reporting a gambling problem (1.9%), more than half engaged in free fantasy leagues and approximately half also played in fee-based fantasy leagues. Almost half of female student-athletes with at-risk/probable-problem gambling (0.1%) reported playing free fantasy leagues, and a quarter reported playing fee-based fantasy leagues. Approximately half of individuals with non-problem gambling reported playing fantasy sports for free and less than a quarter reported playing fantasy sports for monetary gain. In the females with at-risk/probable-problem gambling, there was a 35.9% increase in free fantasy sports playing, and a 23.2% increase of fee-based fantasy sports playing. Most males with at-risk/probable-problem gambling (40.9%) were engaged in between two and five fantasy leagues. Males (65.5%) and females (66.7%) with at-risk/probable-problem gambling did not consider fantasy sports a gambling activity
(Martin & Nelson, 2014)	To investigate fantasy sports involvement among college students, and to explore whether fantasy sports participation is associated with gambling problems	Cross-sectional (2012)	College students N = 1556 36% male; 64% female 70% Caucasian	Past-year fantasy sports participation (participation, free or entry fee, types of leagues and number of leagues)	DSM-5 criteria				Overall, 11.5% reported participating in fantasy sports in the past year with 43.5% of these people playing for money. Males were significantly more likely to play fantasy sports, both for no money and for money. Among those who played fantasy sports without money, 14.9% endorsed ≥ 1 DSM-5 criteria and the rate was higher for female players (26.7%) than male players (11.8%). Among those played fantasy sports for money, 26.9% endorsed ≥ 1 DSM-5 criteria and the rate was higher for male players (27.8%) than female players (16.7%). Fantasy participants (regardless of whether they played for money) were over five times more likely to endorse ≥ 1 DSM-5 GD criteria, and those who participated in fantasy sports for money were significantly more likely to experience one or more gambling-related problems. Male fantasy participants (regardless of whether they played for money) were almost two times more likely to endorse ≥ 1 DSM-5 GD criteria, and males who participated in fantasy sports for money were nearly three times more likely to experience one or more gambling-related problems. Female fantasy participants (regardless of whether they played for money) were almost three times more likely to endorse ≥ 1 DSM-V GD criteria
(Martin et al., 2016)	To examine differences in past year gambling, gambling-related problems, and fantasy sports gambling among college students based on their athlete status (i.e., D1 athlete; club-intramural-recreational (CIR) athlete; and non-athlete (NA))	Cross-sectional (2014)	College student-athletes and non-athletes N = 692 Mage = 20.4 (SD = 1,5 years) 42.6% male; 57.4% female 64.6% Caucasian	Fantasy sports participation and fantasy sports gambling	DSM-5 criteria	Gambling Quantity and Perceived Norms Scale			Fantasy sports players were more likely to report gambling compared to non-players. Being male was the only significant statistical predictor of gambling-related problems, and fantasy sports players were more likely than non-players to experience gambling-related problems. Being male was the only significant statistical predictor of fantasy sports playing, and students with gambling-related problems were more likely to play fantasy sports than those without gambling problems. Fantasy players with gambling-related problems were significantly more likely to gamble on fantasy sports than those without problems
(Martin et al., 2017)	To examine associations between fantasy sports participation (season-long and DFS), gambling frequency, and symptoms of problem gambling	Cross-sectional (2016)	College students N = 941 Mage = 19.8 (SD = 1.4 years) 30.3% male; 69.7% female 68.9% Caucasian	Past-year season-long fantasy sports participation and gambling	DSM-5 criteria	Gambling and gambling frequency: assessed via a single item from the GQPN			Participants who paid an entry fee/deposit to play fantasy sports gambled more frequently than those who did not (*p* < .001). Those who played fantasy sports gambled more frequently than those who did not play fantasy sports (*p* < .001), and individuals who played DFS gambled more frequently than those who only played season-long fantasy (*p* < .01). Among the total sample, 4.9% endorsed one or more DSM-5 GD criteria; people who gambled who played fantasy sports of any kind endorsed more criteria than those who did not play fantasy sports (*p* < .001), and people who gambled who played DFS endorsed more criteria than those who only played season-long fantasy (*p* < .01)
(McCarthy et al., 2018)	To analyze the difference by age and gambling risk status based on gambling frequency, gambling type, reasons for preferring some forms of gambling and women’s perceptions of harm associated with gambling products	Cross-sectional (March—May 2017)	Australian women N = 509 100% female	Past-year engagement	PGSI	Gambling behavior: preferences, perceptions frequency, product use, perceptions of harm			Gambling risk status was associated with frequency of gambling on four forms (electronic gambling machines, horse betting, casino and sports betting) (*p* < .001). Overall, 122 participants reported betting on sports, and over half were from the younger group (50.8%). Those participating in sports betting were more likely to be classified as having at-risk (low or moderate) or problem gambling (*p* < .001). Women who preferred sports betting described it as easy to access and a form of gambling where people could win a lot of money. Females who did not gamble considered sports betting to be more harmful than those with low-risk gambling (*p* = .02), moderate-risk gambling (*p* = .05), and problem gambling (*p* = .03). Younger women were more likely to perceive sports betting as harmful
(McCormack et al., 2013)	To examine participation in online gambling activities and the relationship of these forms of gambling with problem gambling	Cross-sectional (January 2010—May 2010)	People who gambled online recruited from different international gambling forums (30) and gambling websites (2) N = 975 Mage = 34.7 (SD = 13.9 years) years 81.6% male; 18.4% female Caucasian (86.9%), majority from the United Kingdom (51.6%) and United States (33.1%)	Past-year engagement	PGSI	Frequency and duration of gambling sessions, motivations for online gambling, and engagement in multi-gambling			Overall, 14% of participants were identified as having problem gambling (71.7% male, 28.3% female). The mean age of those with problem gambling was 34.6 years (SD = 10.6). Among online activities, 21.1% reported sports betting. For offline gambling, 5.4% reported participating in sports betting ‘most days’. For people with problem gambling, sports betting was the most frequently reported online activity (34.8%), while only 12.2% reported participating in sports betting ‘most days’ offline. In the sample, 30.6% engaged in multiple forms of gambling online, and these individuals were more likely to have at-risk or problem gambling (*p* < 0.001)
(Newall et al., 2021)	To identify which types of people who bet on sports are most likely to use novel gambling products called ‘custom sports bets’ (CSBs) and whether people with gambling problems are more or less likely to use CSB products, whether illusions of control biases are more prevalent among those who use CSBs, whether people who bet on sports who use CSB products experience more gambling-related harm, and how engagement with CSB products varies with sports-betting levels of gambling consumption	Cross-sectional	People who best online on sports/horse racing N = 789 Mage = 35.40 (SD = 10.86 years) 67.3% male; 32.7% female	Past-year engagement	PGSI	Illusion of control subscale of GRCS SGHS CSPG Ad-hoc instrument included questions about custom bet products			Overall, 16.0% of people engaging in custom sports betting (CSB) had current problem gambling (PGSI score 8), compared to 6.7% of those who did not use CSB products. PGSI scores were significantly higher amongst people using CSBs (*p* < 0.001). People using CSBs had higher illusions of control (*p* = 0.031), experienced more gambling-related harms (*p* < .001), and had higher scores in the consumption screen for problem gambling (*p* < 0.001)
(Nweze et al., 2020)	To identify whether young Nigerian individuals who regularly engage in online sports betting have difficulties with decision-making	Cross-sectional	People who bet on sports and those who do not N = 78 Non-sports-betting: Mage = 22.24 (SD = 3.97 years) 36 males; 6 females Sports-betting: Mage = 22.69 (SD = 3.64 years) 33 males; 2 females	Past year engagement	G-SAS			IGT, The colour-shape-shifting task, BIS	Most people who bet on sports reported gambling-related behavioral problems in the mild (*n* = 23) or moderate range (*n* = 11), with only one participant falling into the extreme and severe symptom category on the G-SAS. Between people who bet on sports and those who did not, no significant differences were reported in the number of chosen disadvantageous decks nor the outcomes on the IGT. Regarding cognitive flexibility, error rates differed significantly between groups (*p* = .02), where people who bet on sports (13%) committed more errors than those who did not (8%). No substantial correlations between performance measures and clinical variables were observed
(Orlowski et al., 2020)	To compare different gambling types with respect to cognitive distortions and disordered gambling	Cross-sectional (May 2016) data taken from larger longitudinal study (MIGUEL project)	Vocational school students N = 309 Mage = 20.13 (SD = 2.52 years) 87% male; 13% female 67% of participants report immigration	Past-year engagement	Stinchfield self-reporting questionnaire of gambling-related problems	GBQ. Type of gambling activities			Sports betting was identified as the only statistical predictor for problematic gambling (OR = 1.91), but the level of significance was lost when adjusting for cognitive distortions
(Phillips et al., 2013)	To explore the relationship between increased involvement and access to a range of gambling products and risk of problem gambling	Cross-sectional	Sample 1 undergraduate psychology students N = 464 Mage = 20.40 (SD = 4.58 years) 133 males; 329 females; 2 unspecified Sample 2 community respondents N = 1,141 Mage = 37.7 (SD = 12.79 years) 490 males; 646 females	Past engagement	SOGS PGSI	Number of gambling activities			In community respondents, engagement in sport wagering was a significant statistical predictor of gambling problems (*p* < 0.001)
(Quilty et al., 2014)	To examine the relationship between involvement in gambling, gambling harm and problem gambling and to evaluate the contribution of related gambling activity indicators in statistically predicting harmful or problematic gambling	Cross-sectional	Community and clinical gambling samples (psychiatric outpatients) N = 503 Community gambling sample (N = 228) Mage = 40.47 (SD = 13.12 years) 115 males; 113 females Clinical gambling sample (N = 275) Mage = 43.02 (SD = 11.58 years) 100 males; 175 females	Past engagement	CPGI	Harmful and problem gambling assessed by some items of the PGSI	DSM–IV Axis I pathology		Overall, 90 participants in the community sample and 30 in the clinical sample met the criteria for lifetime pathological gambling. Gambling frequency, duration, and expenditures statistically predicted the presence versus absence of harmful gambling. Individuals gambling at a greater frequency were more likely to be classified as experiencing harm from gambling. Duration of gambling appeared to contribute little practical information in conjunction with gambling frequency in individuals who gamble more frequently having a greater likelihood of developing problem gambling symptoms. For sports betting, the gambling expenditure cutoff for harmful gambling was $42.5 to $65 Canadian per month (sensitivity .52 to .66; specificity .68 to .85)
(Rhind et al., 2014)	To examine the extent of gambling and associated corruption among athletes in the United Kingdom	Cross-sectional	Athletes N = 1,049 53.1% males; 46.9% females Competitive levels: international/national (5.3%), country (32.2%), club competitions (62.5%)	Past gambling on sporting events	PGSI			Involvement in corruptive practices	Overall, 61.3% of males and 21.6% of females reported gambling on at least one sporting event over the past year, with males gambling more frequently than females. By group, 15% of males and 4.6% of females were identified as having low-level gambling, 13.7% of males and 1.5% of females were classified as having moderate level gambling and 9.2% of males and 1.1% of females as having problem gambling. Male respondents reported engagement in corruptive practices at a higher level than females. Athletes competing at the international/national level (8.3%) were more likely to have been asked to provide inside information as well as to have been asked to influence the outcome of a game (8.3%)
(Richard et al., 2019)	To explore the following gambling trends over 12 years among student-athletes: gambling participation, problem gambling, origins of gambling behaviors and attitudes towards sports wagering	Four cross-sectional studies (2004, 2008, 2012, and 2016)	College student-athletes 2004 (N = 20,587): 62% men, 38% women, 75% Caucasian 2008 (N = 19,942): 62% men, 38% women, 72% Caucasian 2012 (N = 22,935): 57% men, 43% women, 77% Caucasian 2016 (N = 22,388): 61% men, 39% women, 70% Caucasian	Past engagement	Problem Gambling assessed by DSM-IV-TR criteria	GAQ Gambling Knowledge and Attitudes Type of sport played, division in which they competed (Division I, II and III) Gambling experiences			Sports wagering appears to be a frequent activity among men. Rates of betting on one’s own team decreased over time, with the highest rates being in 2008 (*p* < .001). However, no significant changes were reported for betting on another college team. Participation in fantasy leagues involving entry fees and prize money significantly increased from 17% in 2008 to 20% in 2016. Females reported lower rates of gambling participation compared to men, and the rates of sports wagering decrease over time. In 2016, 39% of men and 20% of women reported believing that sports wagering was acceptable as long as they were wagering on a sport in which they do not participate. Similarly, in 2016, 49% of men and 31% of women perceived sports wagering as a harmless pastime
(Roderique-Davies et al., 2020)	To measure the cue-induced urge effect of embedded gambling promotions in televised sports among student athletes and non-athletes	Cross-sectional	Student athletes and non-athletes N = 60 Mage = 22.67 (SD = 4.01 years) 35 males; 25 females	Past gambling on sporting events	PGSI	GUS			Student athletes reported a significantly higher risk of gambling problem scores (*p* < 0.001) and reported significantly higher urges to gamble (*p* = 0.001) compared to non-athlete students. Among student athletes, 30% engaged in online sports betting compared to 16.67% of non-athlete students. Student athletes were also more likely to gamble on a weekly basis (30% vs 6.67%)
(Russell et al., 2019a)	To examine demographic, behavioral and psychological factors specific to sports betting	Cross-sectional	General population N = 1147 Mage = 41.17 (SD = 14.50 years) 66.5% male; 33.5% female 83.5% born in Australia	Expenditure, frequency, proportion of sports bets based on land-based venues, and type of competitions Payment methods Age of first sport betting. Frequency of watching sporting contests on which they bet Involvement in fantasy sports	SB-PGSI (adapted from the PGSI)	Frequency of betting on six forms of gambling. Number of bets placed in a typical day of betting. Motivations for gambling. GOES. GUS. GBQ	Alcohol use (4 item of CAGE)	BSCS	Overall, 394 (34.3%) respondents were classified as having non-problem gambling, 239 (20.8%) as having low-risk gambling, 209 (18.2%) as having moderate-risk gambling and 306 (26.7%) as having problem gambling. Potential risk factors for having moderate-risk or problematic sports betting included: a higher monthly sports betting expenditure, higher gambling urges, alcohol-use-related concerns, and lower self-control. The most significant statistical predictors of moderate-risk or problematic sports betting included: being motivated by money, experiencing greater gambling urges, having greater erroneous cognitions, alcohol-use-related concerns, and lower self-control. Those at higher risk of sports-betting-related problems were younger, spoke a language other than English as their main language, and had lower self-control
(Russell et al., 2019b)	To examine the association between micro-event betting and problem gambling and to determine the demographic, behavioral and psychological characteristics of people who bet on micro-events	Cross-sectional (2016)	People who bet on sports N = 1813 Mage = 35.3 (SD = 12.6 years) 68.9% male; 31.1% female	Percentage of people who bet on sports by: land-based venues, the final outcome, key events and on micro-events within the match Frequency of watching sports and of seeing or hearing gambling advertisements. Use of sports-betting promotions Expenditure	PGSI	Frequency of gambling on six activities Number of days they bet per month Perceived social norms related to gambling Number of accounts with different wagering operators		BISBrief	In the current sample, 21% reported sports betting more than once a week, 19.7% weekly, 17% 2 to 3 times a month, 18.5% monthly, and 23.9% less than once a month. Overall, 46.8% of participants scored as having problem gambling (non-problem 19.5%; low-risk 16.2%; moderate risk 17.6%). 36.8% reported placing at least 1% of their sports bets on micro-events in the previous year. Those who reported betting on micro-events scored higher on impulsivity and 77.8% were classified as having problem gambling in contrast to 28.7% of those who did not bet on micro-events. People who bet on micro-events were younger, were more likely to be single, had a higher level of education, engaged in more forms of gambling, bet on sports more frequently, bet on a greater number of different sports, placed more bets per sports-betting day, placed more of their bets via telephone, perceived that their peers bet more frequently on sports, reported a lower frequency of exposure to gambling-related advertising, and used promotions more frequently. In a regression model, statistical predictors of betting on micro-events were: betting on a higher number of different sports, placing more bets per sports-betting day, placing more of their bets by telephone, and having problem gambling. Placing a higher proportion of bets on micro-events was significantly associated with speaking English as a main language at home, having problem gambling, placing a lower proportion of bets via the internet, and placing one’s last impulse bet more recently. Significant statistical predictors of placing a higher proportion of sports bets on micro-events included having problem gambling, having more accounts with different operators and placing a lower proportion of bets via the internet
(Russell et al., 2019)	To examine potential demographic, behavioral, marketing, and normative risk factors for sports betting among all levels of gambling-related problems	Cross-sectional (July—September 2016)	People who bet on sports N = 1813 Mage = 35.3 (SD = 12.6 years) 68.9% male; 31.1% female	Frequency, type of sport, onset, number of accounts with different operators, expenditure, number of days betting per week, average of bets per days, method used, percentage of bet on the final score, and on micro-events Frequency of watching sports live and of seeing or hearing advertisements and promotions for sports betting, and number of promotions used. Peer norms for sports betting	PGSI			BISBrief	People with moderate-risk and problem gambling reported significantly higher sports-betting expenditures than those with non-problem gambling. Compared to people with non-problem gambling, those with problem gambling placed bets on more days per month and higher proportions of bets during the match and on micro-events within the match. People with problem gambling reported that their peers bet a significantly higher amount of money. People with low-risk, moderate-risk and problem gambling reported using a higher number of different types of promotions and had higher impulsiveness scores compared to people with non-problem gambling
(Valleur et al., 2016)	To verify the validity of classifying people who gamble based on the Pathways Model (i.e., behaviorally conditioned, emotionally vulnerable, and antisocial/impulsivity) in a large cohort of people with problematic gambling	Cross-sectional (2009)	GD N = 372 Behaviorally conditioned group (N = 162), Mage = 43.5 (SD = 12.6 years), 72.8% male; 67.2% female Emotionally vulnerable group (N = 111), Mage = 44.1 (SD = 13.1 years) 67.6% male; 32.4% female Antisocial/Impulsivity group (N = 99), Mage = 41.8 (SD = 11.6 years) 83.8% males; 16.2% females	Past engagement in sports betting	DSM-IV criteria SOGS	GABS-23 Gambling activities, monthly gambling expenditure, maximum wagering in a single day, age of initiation into gambling and family history of problem gambling	MINI to ecplore axis-I psychiatric disorders, plus current risk of suicide and antisocial personality disorder	WURS-C. TCI-125	Three subgroups of people with problem gambling were identified: behaviorally conditioned (not fitting in the other groups), emotionally vulnerable (reporting at least one episode of anxiety or depression before having a gambling problem) and antisocial/impulsivity (reporting antisocial personality disorder or high novelty-seeking). The antisocial/impulsivity gambling group preferentially chose semi-skillful gambling such as sports gambling, whereas the emotionally vulnerable group was more likely to gamble on games of chance. The antisocial/impulsivity group had a greater proportion of co-occurring disorders: 53.5% had a history of mood disorders, 36.4% a history of anxiety disorders, 50.5% a history of addictive disorders other than gambling, and 9.1% a history of a psychotic condition. Sports betting was preferred by 12.4% of the antisocial/impulsivity group, versus 8.3% of the emotionally vulnerable group and 10.8% of the behaviorally conditioned group
(Wang et al., 2021)	To investigate college students’ sports-betting intentions and behaviors using the theory of planned behavior (TPB) and whether problem-gambling severity moderates the relationships between attitudes toward sports gambling (ATSG), subjective norms (SN), perceived behavioral control (PBC), sports gambling intention (SGI), sports gambling behavior (SGB), and problem gambling	Cross-sectional	College students with recreational gambling (n = 217) and problem gambling(n = 117) N = 334 Mage = 21.0 (SD = 2.37 years) 67.7% males; 32.3% females 82.3% Caucasian	Attitude toward sports gambling (ATSG), subjective norms (SN), perceived behavioral control (PBC), sports gambling intention (SGI), sports gambling behavior (SGB)	Problem gambling				Overall, 81.1% reported engagement in some form of gambling, with 72.8% reporting sports gambling. The intention to bet on sports was statistically predicted by attitude (*p* < 0.001) and subjective norms (*p* < 0.001). Actual sports-betting behaviors were predicted by intention (*p* < 0.001) and perceived behavioral control (PBC) (*p* = 0.02). Both attitudes and subjective norms, directly and indirectly, influenced college students’ sports-betting behaviors (*p* < 0.001). In the lower-risk gambling group, sport gambling intention was mostly statistically predicted by attitude (*p* < 0.001), followed by subjective norms (*p* < 0.001). Within this group, sports betting was statistically predicted by intention (*p* < 0.001) and PBC (*p* = 0.016). In the higher-risk gambling group, sports-betting intention was statistically predicted by subjective norms (*p* < 0.001) and attitudes (*p* = 0.004), but not by PBC. Finally, the problem-gambling group’s sports-betting behavior was statistically predicted by both intention (*p* < 0.001) and PBC (*p* = 0.02). For both groups, the indirect effects of attitude and subjective norms via intention on behavior were significant. The role of attitude toward sports gambling was more influential in predicting the gambling behaviors of the lower-risk gambling group (*p* = 0.04)
(Wardle et al., 2021)	To examine changes reported by people who regularly bet on sports in their gambling behaviors during the COVID-19 lockdown period and to explore whether such changes are related to gambling harms	Cross-sectional (July 2020)	People who bet on sports, N = 3,866 Men: Non-problem gambling (n = 2096), Low-risk gambling (n = 564), Moderate-risk gambling (n = 289), Problem-gambling (n = 135). Regular sports betting: Non-problem gambling (64.7%), Low-risk gambling (19.3%), Moderate-risk gambling (10.7%), Problem gambling (5.4%) Women: Non-problem gambling (n = 574), Low-risk gambling (n = 124), Moderate-risk gambling (n = 59), Problem gambling (n = 25). Regular sports betting: Non-problem gambling (71.2%), Low-risk gambling (16.6%), Moderate-risk gambling (8.3%), Problem gambling (3.9%)	Past engagement in sports betting	PGSI	Gambling attitudes, awareness of gambling marketing, experiences of gambling harm, Gambling behaviors before, and during, the initial stages of the COVID-19 pandemic		COVID-19 health and lifestyle experiences	Before the COVID-19 lockdown, online sports betting was the most commonly reported gambling activity (78.7% men, 61.4% women). During the initial COVID-19 lockdown, 17.3% of men and 16.5% of women started gambling on at least one new form of gambling; 29.8% of men and 33.4% of women stopped all gambling during the initial COVID-19 lockdown; and 31.3% of males betting on sports and 30.3% of females betting on sports increased their frequency of gambling on at least one activity during the initial COVID-19 lockdown. 5.4% of males betting on sports and 3.9% of females betting on sports experienced problem gambling (PGSI score ≥ 8) during the initial COVID-19 lockdown. A further 10.7% of men and 8.3% of women experienced moderate-risk gambling (PGSI score of 3–7). Men were more likely to experience problem gambling if they were younger (under 35 compared with over 55 years) (AOR = 5.24), had lower rather than higher wellbeing scores (AOR = 2.23), started a new form of gambling during lockdown (AOR = 2.50), or had changed their level of spending on gambling. For women, age (being under 35 years), lower rates of wellbeing, shielding status, and increases in gambling frequency were all associated with moderate-risk or problem gambling
(Weiss & Loubier, 2010)	To determine what types of gambling are the most prevalent among non-athletes, current athletes, and former athletes experiencing symptoms of problem gambling	Cross-sectional	Current athletes, former athletes, and nonsathletes N = 860 Mage: Nonathletes = 35.03 years; former athletes = 34.86 years; current athletes = 32.60 years 15 participants of each gender were included in each of the three athletic status groups Caucasian = 54%, Hispanic/Latina = 23%, African American = 14%, Asian American = 4%, Native American = 2%, and 1% Other	Past engagement in sports betting	SOGS	Different types of gambling behaviors			Former athletes had the highest frequency of involvement in sports gambling (70.0%), followed by current athletes (40.0%) and non-athletes (20.0%). In athletes (current and former), there was a relationship between the sport that they played and the sport on which they gambled (*p* < .001). In general, former athletes were more likely to participate in skill-based forms of gambling such as sports gambling and poker, whereas non-athletes were more likely to engage in forms of gambling that are based predominantly on chance

*AUDIT *Substance use: alcohol use disorders identification test; *BDI* beck depression inventory, *BIS* barratt impulsiveness scale, *BIS-Brief* barratt impulsiveness scale-Brief, *BSCS* brief self-control scale, *BSSS* brief sensation seeking scale, *CEPER* ceper exploration personality questionnaire, *CPGI* canadian problem gambling index, *CSPG* consumption screen for problem gambling, *DFS* daily fantasy sports, *DSM* diagnostic statistical manual, *DSM–IV–TR*: DSM text revision, *GABS*-23: gambling attitudes and beliefs survey—revised version, *GAQ* gambling activities questionnaire, *GBQ* gamblers' beliefs questionnaire, *GD *gambling disorder, *GMS *gambling motivation scale, *G-SAS* the gambling symptom assessment scale, *GOES* gambling outcomes expectancies scale, *GQPN* gambling quantity and perceived norms Scale, *GRCS* gambling-related cognition scale, *GUS* gambling urge scale, *HAM-A* hamilton anxiety scale, *IGT* iowa gambling task, *K6* kessler psychological distress scale, *Mage* mean age; MH5: Mental Health 5 Item Scale; MINI: Mini International Neuropsychiatric Interview; MSSFP: motivational scale for Fantasy Football Participation, *NODS* diagnostic screen for gambling disorders loss of control, lying, and preoccupation “CLiP”, PGSI:

#### Samples

Most (*n* = 52) studies involved adults from the general population. Seven studies included clinical samples with alcohol use disorders (Bodor et al., 2018) or GD (Barrera-Algarín & Vázquez-Fernández, 2021; Estévez et al., 2017; Håkansson et al., 2017; Jiménez-Murcia et al., 2021; Quilty et al., 2014; Valleur et al., 2016). Ten studies included samples of college students and/or athletes (Grall-Bronnec et al., 2016; Marchica & Derevensky, 2016; Martin & Nelson, 2014; Martin et al., 2016, 2018; Phillips et al., 2013; Richard et al., 2019; Roderique-Davies et al., 2020; Wang et al., 2021; Weiss & Loubier, 2010).

#### Assessment of Sports Betting and GD

Most studies assessed past-year engagement in sports betting, online sports betting, or fantasy sports. Some studies assessed engagement in sports betting during average weeks or months. Other factors assessed included: sports gambling expenditures, frequency of sports lottery purchasing in a week, time commitments to daily sports lottery-related activities, types of sports lottery purchased, channels used to bet on sports, devices used, engagement with in-play betting, gambling activities participation (frequency, seasonal fantasy sports betting, daily fantasy sports betting, and sports betting in general), monthly sports-betting expenditures over the past year, frequency of sports-betting behaviors, proportion of sports bets in land-based venues, via the internet and via telephone calls, payment methods used for sports betting, and age of first sport betting.

The psychometric instruments used to assess GD were heterogeneous and are described in Table 2. The most frequently used measures to assess for the presence of problem gambling and/or GD were PGSI (Ferris & Wynne, 2001), DSM-IV criteria (American Psychiatric Association, 2010) and DSM-5 criteria (American Psychiatric Association, 2013).

**Table 2:** Psychometric instruments used to assess GD

Instrument full name	Abbreviation	Reference
Problem Gambling Severity Index	PGSI	Ferris, J., & Wynne, 2001
DSM diagnostic criteria for GD	–	American Psychiatric Association, 2013
South Oaks Gambling Screen	SOGS	Lesieur & Blume, 1987
Lie/Bet questionnaire based on two DSM-IV criteria for pathological gambling	–	Johnson et al., 1997
Scale of Assessing Problem Gambling	SAPG	Li et al., 2012
Gambling Symptom Assessment Scale	G-SAS	Kim et al., 2009
Stinchfield self-reporting questionnaire of gambling-related problems	–	Stinchfield, 2002
Canadian Problem Gambling Index	CPGI	Ferris, J., & Wynne, 2001
Sports Betting Problem Gambling Severity Index	SB-PGSI	Russell et al., 2019a, 2019b Adapted from the original PGSI by Ferris, J., & Wynne, 2001

#### Risk Bias Assessment Results

As depicted in Table 3, most studies had global ratings corresponding to strong and moderate quality, almost in equal proportions (strong *n* = 24; moderate *n* = 25), and only 5 studies were rated as having a weak global quality.

**Table 3:** Risk bias assessment and results

1st Author, year	Selection bias	Study design	Data collection methods	Appropriate statistical tests	Global rating
(Baggio et al., 2018)	3	1	1	1	2
(Barrera-Algarín & Vázquez-Fernández, 2021)	3	1	1	2	2
(Bodor et al., 2108)	3	1	1	2	2
(Brevers et al., 2021)	2	3	1	2	2
(Cooper et al., 2021)	2	1	1	1	1
(DiCicco-Bloom & Romer, 2012)	2	1	1	1	1
(Dwyer et al., 2018)	2	3	1	1	2
(Estévez et al., 2017)	1	1	1	2	1
(Gainsbury et al., 2019)	2	2	1	1	1
(Grall-Bronnec et al., 2016)	3	2	1	1	2
(Granero et al., 2020)	1	1	1	1	1
(Håkansson, 2020)	3	2	1	2	2
(Håkansson et al., 2017)	3	1	2	2	2
(Håkansson & Widinghoff, 2020)	3	2	1	1	2
(Hing et al., 2019)	2	3	1	2	2
(Hing & Haw, 2009)	3	3	1	2	3
(Hing et al., 2018a, 2018b)	1	1	1	1	1
(Hing et al., 2017)	3	1	1	1	2
(Hing et al., 2016)	3	1	1	2	2
(Hing et al., 2016)	2	2	1	2	1
(Hing et al., 2017)	2	3	2	1	2
(Hing et al., 2018a, 2018b)	2	1	1	1	1
(Hing et al., 2015a)	2	2	2	1	1
(Hing et al., 2015a, 2015b)	2	3	2	2	2
(Holtgraves, 2009)	3	1	1	1	2
(Jiménez-Murcia et al., 2021)	1	1	1	1	1
(E. Li et al., 2020)	2	2	1	1	1
(H. Li et al., 2015)	1	1	1	1	1
(Lopez-Gonzalez et al., 2018)	2	2	1	1	1
(Lopez-Gonzalez et al., 2020)	2	2	1	1	1
(Lopez-Gonzalez et al., 2019)	2	2	1	2	1
(Lopez-Gonzalez & Griffiths, 2021)	1	2	1	2	1
(Marchica et al., 2017)	2	1	1	1	1
(Marchica & Derevensky, 2016)	2	1	1	3	2
(Martin & Nelson, 2014)	2	1	1	1	1
(Martin et al., 2016)	2	2	1	1	1
(Martin et al., 2017)	3	2	1	1	2
(McCarthy et al., 2018)	3	3	1	1	3
(McCormack et al., 2013)	3	2	1	2	2
(Newall et al., 2021)	2	2	1	1	1
(Nweze et al., 2020)	2	3	1	1	2
(Orlowski et al., 2020)	3	3	1	1	3
(Phillips et al., 2013)	3	3	1	1	3
(Quilty et al., 2014)	3	1	1	1	2
(Rhind et al., 2014)	3	2	1	2	2
(Richard et al., 2019)	3	1	1	2	2
(Roderique-Davies et al., 2020)	2	3	1	2	2
(Russell et al., 2019a, 2019b)	1	2	1	1	1
(Russell et al., 2019a, 2019b)	1	1	1	1	1
(Russell, Hing, Li, et al., 2019)	1	1	1	2	1
(Valleur et al., 2016)	3	1	1	1	2
(Wang et al., 2021)	2	3	3	1	3
(Wardle et al., 2021)	2	1	1	1	1
(Weiss & Loubier, 2010)	3	2	1	2	2

*N/A* not applicable, 1 strong, 2 moderate, 3 weak

### Sports Betting and Sociodemographic Characteristics

Sports betting was highly associated with sex, with males being more likely to participate in sports-betting and fantasy-sports leagues (Håkansson et al., 2017; Marchica & Derevensky, 2016; Martin & Nelson, 2014; Richard et al., 2019). Differences have also been reported between males and females in the association between sports betting and substance use. While in the case of females, this association seems to be relatively weak, for males it appears to be particularly strong (Baggio et al., 2018). Regarding age, Marchica et al. (2017) observed that students aged 16–19 years were more likely to exhibit features of problem gambling when they engaged in sports-related gambling regularly, compared with younger adolescents aged 13–15 years.

Numerous attempts have also been made to define a phenotype related to sports betting by considering multiple sociodemographic variables together. Findings suggest that individuals engaging in sports betting are more likely to be male, single, and younger than non-sports-betting individuals (Cooper et al., 2021). They are also more likely to be college-educated, have full-time jobs with higher salaries, and report betting a higher percentage of their monthly incomes (Cooper et al., 2021). In a predictive model of sports betting delineated by DiCicco-Bloom and Romer (2012), non-Hispanic Black ethnicity was a significant statistical predictor of sports betting, as well as having a friend who gambles or approves of one’s gambling. Jiménez-Murcia et al. (2021) reported that being male, younger, engaging in more frequent sports betting, and presenting higher impulsivity and lower self-directedness levels, among other measures, were factors increasing the risk of online sports betting. The highest frequency of sports betting was observed in males, with high social status and low education levels also being implicated (Jiménez-Murcia et al., 2021).

Granero et al. (2020) identified two latent clusters of sports betting. Cluster 1 included individuals most involved in online sports betting. This group was characterized by being younger and unmarried, having a lower socioeconomic status, engaging in gambling activities early in life, reporting higher gambling-related debts, and reporting more substance-use disorders, worse psychopathology and dysfunctional personality traits. Cluster 2 included individuals less involved in online sports betting. These individuals were older, mostly married or with a stable partner, and were of higher socioeconomic status.

### Sports Betting and Gambling-Related Variables

#### Problem and Disordered Gambling Severity

A significant association between sports betting and problem gambling was reported in most studies (Baggio et al., 2018; Lopez-Gonzalez et al., 2020; Nweze et al., 2020; Russell et al., 2019a, 2019b). Cooper et al. (2021) observed that, compared to non-sports-betting individuals, those betting on sports were more likely to report problem gambling, and this could be related to group differences in attitudes towards gambling, cognitive distortions, gambling motivations or the number of gambling activities performed. Similarly, Gainsbury et al. (2019) reported a positive correlation between a higher frequency of online sports betting and problem gambling. In this vein, Bodor et al. (2018) reported that participants with alcohol use disorder who reported a higher frequency of sports betting were more likely to exhibit at-risk/problem gambling or to have GD. In DiCicco-Bloom and Romer’s study (2012), symptoms of GD including tolerance and worry were statistically predictive of sports betting, while withdrawal and subjective loss of control were not.

Not all studies identified clear associations between sports betting and problem gambling. For example, Håkansson and Widinghoff (2020) highlighted associations between problem gambling and the combination of online casino gambling and live sports betting. However, this association was not significant for live-sports betting alone.

Some studies were indicative of a relationship between sports betting and GD severity, with sports betting being a statistical predictor of problem gambling (Hing et al., 2016; Orlowski et al., 2020; Phillips et al., 2013; Russell et al., 2019a, 2019b). Others have reported that problem-gambling severity was the strongest statistical predictor of the frequency of sports betting (). Individuals participating in fantasy sports, including those playing fantasy sports for money, were more likely to report problematic gambling compared to those without such engagement (Lopez-Gonzalez et al., 2019; Martin et al., 2016, 2018), (Martin & Nelson, 2014). Regarding adolescents, the strongest statistical predictor of at-risk gambling for individuals aged 13–15 years was regular participation in daily fantasy sports (Marchica et al., 2017).

#### Other Gambling Activities and Gambling Frequency

Participation in sports, lottery, and slot-machine gambling have been found to be statistical predictors of sports betting (DiCicco-Bloom & Romer, 2012). Marchica et al. (2017) found that at-risk gambling was statistically predicted by all forms of sport-relevant gambling activities. Other gambling-related variables considered potential risk factors for online sports betting included a higher frequency of sports betting in general and a greater presence of negative attitudes towards gambling (Hing et al., 2017).

In Estévez et al.’s study (2017), online sports betting was associated with higher wagers compared to non-sports online betting and offline gambling. In this vein, Håkansson and Widinghoff (2020) reported that people who combined online casino gambling and live sports betting were more likely to report over-indebtedness, which did not occur in the case of live-sports betting alone. Gambling-related factors, such as betting on a greater number of different sports, more frequent exposure to promotions, and more positive attitudes towards them were statistical predictors of betting on micro-events within sporting events (Russell et al., 2019a, 2019b) or greater intended frequency of sports betting ().

### Sports Betting and Psychopathology

In Gainsbury et al.’s study (2019), psychological distress was associated with a higher frequency of sports betting. Similarly, Hing et al. (2017) noticed that problematic online sports betting, as compared to non-problematic online sports betting, was associated with more psychological/emotional distress. Likewise, the use of alcohol and other substances, as well as the presence of other behavioral addictions, have been reported in different studies, with percentages being substantial, ranging from 22.1% to 71.2% among individuals engaged in sports betting (Bodor et al., 2018; Granero et al., 2020; Hing et al., 2017).

### Sports Betting and Personality Tendencies

Higher levels of impulsivity, sensation-seeking and positive and negative urgency have been reported among people betting on sports (Cooper et al., 2021). Among the multiple factors analyzed, impulsivity (and more specifically negative urgency) was the measure that most distinguished non-sports-betting and sports-betting groups. DiCicco-Bloom and Romer (2012) observed that one of the most significant statistical predictors of sports betting was sensation-seeking.

When analyzing impulsive in-play bets, Hing et al., (2018a, 2018b) reported that these types of wagers were statistically predicted by problem gambling and greater buying impulsiveness, among other factors. In this vein, Hing et al., (2018a, 2018b) observed that people who gambled on sports immediately before a match were characterized by higher levels of impulsivity, which is in concordance with the study by Jiménez-Murcia et al., (2021).

In reference to persistence as a personality feature, mixed results have been reported. Whereas in some studies it was reported that online sports betting was linked to higher levels of persistence, compared to non-sports online betting and offline gambling (Estévez et al., 2017), others have found that greater sports-betting frequency associated with lower persistence (Jiménez-Murcia et al., 2021). Finally, it has been suggested that, in the case of sports betting, other relevant statistical predictors of problem-gambling severity include lower self-directedness and greater psychopathological distress (Jiménez-Murcia et al., 2021).

## Discussion

The increased participation in sports betting facilitated by online-gambling modalities has led to an increase in the number of sports-betting patients treated in behavioral addiction units (Mestre-Bach et al., 2022). A significant gap in the literature has involved conceptualizing OSB as simply another form of online gambling without attending to the potentially specific risks associated with online or offline forms of sports betting. The present systematic review was conducted to obtain clinically relevant information on sociodemographic characteristics, gambling-related variables, co-occurring psychopathology and personality features related to sports betting.

Most studies included in this systematic review were published within the last decade, underlining the novelty of research in this area. The number of studies reviewed is sufficient to establish sociodemographic and clinical profiles of people who bet on sports who may be at an increased risk of problem or disordered gambling. The present study has synthesized research findings revealing distinctive characteristics of individuals reporting sport betting who suffer from gambling problems.

Gambling problems related to sports betting are associated with being younger, male, single, and college-educated, and experiencing a faster evolution of GD which is often associated with higher levels of severity compared to other subtypes of individuals with gambling problems. We also found that sports betting with gambling problems to be associated with impulsivity sensation-seeking, psychological distress, frequent use of alcohol or other substances and other (non-GD) behavioral addictions. In this sense, sports betting is linked to specific personality profiles with certain biologically based predispositions (e.g., impulsivity, compulsivity and sensitivity to reward and punishment) that may promote vulnerability to addictions. Such an endophenotype may be particularly concerning as it is not only a risk factor for problematic engagement in sports betting, but also a factor that may maintain problematic behaviors across time.

Findings from this systematic review have important implications for prevention and treatment. As sports betting related to problem-gambling risk is linked to younger age, individuals within this subset of the population should be considered a target for preventative actions and programs. As younger individuals with GD are also more likely to drop out of treatment, bolstering the effectiveness of current preventative approaches to decrease the risk of individuals developing problems with gambling is important (Estevez et al., 2021; Ford & Håkansson, 2020; Granero et al., 2020; Jiménez-Murcia et al., 2015). Moreover, as sports-betting advertising has expanded as a form of marketing in sporting events (Bouguettaya et al., 2020; Newall et al., 2019), preventative messages included within these advertising campaigns could help increase awareness of the risks associated with sports betting and knowledge as to when it is important to seek out additional consultation or treatment.

Besides the homogeneity found in the description of people who bet on sports (i.e., male, younger age, single), a recent study identified sports betting with gambling problems to be associated with other distinct features (i.e., earlier onset of gambling activity, higher psychopathological distress, and more dysfunctional personality profile) (Granero et al., 2020). Since this study was conducted in Spain, and most of the studies included within this review were performed in Australia, it will be interesting to increase our efforts in differentiating features linked to sports betting based on cultural backgrounds. This may lead to the development of more specific and effective treatments that are tailored to the needs of people with sports-betting-related GD based on their geographic locations and cultural backgrounds.

Regarding personality, both impulsivity and sensation-seeking have been reported to be frequently present in this profile of individuals with gambling problems (Zilberman et al., 2018) and have been associated with a higher severity or disordered gambling among those meeting the diagnostic criteria (Hing et al., 2018a, 2018b). This may be particularly worrying considering that the types of wagers becoming available via OSB are based on micro-events within sporting events and include in-play bets, which may be particularly attractive to impulsive individuals. This tendency may lead people to spending more money or wagering more frequently than they had initially intended, resulting in various psychological or financial consequences. These findings suggest the importance of the inclusion of features within OSB websites or applications to deactivate certain forms of wagering based on individuals’ preferences or predetermined level of risk. This may be an important aspect to consider in discussions involving regulators and companies providing sports-betting products to an ever-increasing population of people across the globe.

### Limitations and Future Research

The studies included in this review have noteworthy limitations. Most studies (1) were cross-sectional, meaning that causality could not be inferred and therefore longitudinal studies are needed to understand temporal relationship between the measured variables; (2) assessed participants using self-administered instruments, which may be subject to biases associated with social desirability or introspective differences; (3) recruited samples using non-probability online panels, which limits the generalizability of the results and could have promoted greater selection of people who bet online versus offline; (4) included small samples; (5) had unbalanced samples, with very low representation of women; and (6) used different instruments to assess each of the measured variables, with this heterogeneity limiting the generalizability of the results. Further, given the heterogeneity of the samples and their geographic locations, with different legislations in different countries, comparability between the studies is complicated and limited.

Limitations specific to the present systematic review should also be noted. First, only studies in English, French and Spanish were included, which could limit generalizability. Second, in some countries, there is considerable gray literature related to GD, as highlighted by other authors (King et al., 2020), which was excluded in the present systematic review due to the desired empirical rigor of the included articles. Third, although the present review was focused on specific sociodemographic and clinical/psychological variables associated with sports betting, there are other factors, including neurobiological, physiological, and sociocultural variables that may be associated with sports betting that were not included. These are hypothetically important variables that should be considered in future reviews.

Based on the findings included within the present systematic review, future investigations of the following topics should be considered. First, empirical studies focused on fantasy sports (both daily and league-based) and their possible associations with GD should be conducted. There was a paucity of such studies included in this review, and this is an important domain of sports betting worth studying. Second, longitudinal studies across different developmental periods (e.g., adolescence, emerging adulthood, adulthood) and involving vulnerable groups are needed to understand the trajectories or maintenance of sports betting and problem/disordered gambling across time while considering factors that may moderate or mediate such relationships.

### Clinical Implications

Further exploration of the phenotypes related to sports betting, among both adolescents and adults, especially those with problem gambling, is clinically important. Data obtained regarding risk and maintenance factors associated with sports-gambling behaviors should help treatment-development efforts. Likewise, delving into the possible co-occurrence of problem gambling related to sports betting and other disorders may help to understand poor treatment outcomes in these individuals and promote more personalized interventions.

As described in this systematic review, the findings of the studies published to date suggest that people with sports-gambling-related GD have a specific sociodemographic, clinical and personality profile that differentiates them from others with GD. Thus, their youth, the rapid evolution of the problem, the cognitive distortions (minimizing the risk of sports betting and overestimating their skills and knowledge to obtain prizes), as well as the associated personality traits (high impulsivity and novelty seeking), suggest the need to implement tailor-made treatment programs. In addition, all the characteristics mentioned above are associated with higher likelihoods of dropout from treatment programs. Therefore, cognitive restructuring strategies, the implementation of healthy and alternative leisure activities and the use of new technologies (such as serious games) for the treatment of these underlying personality characteristics, but from a motivating and entertaining environment, may be particularly useful.

## Conclusions

Sports betting is becoming increasingly widespread, and a growing number of individuals, both adolescents and adults, are involved in this form of gambling. The present systematic review included studies that explored the sociodemographic and clinical characteristics associated with sports betting. Many studies indicated associations between sports betting and problem gambling. Further, findings indicated that males with high levels of impulsivity reported more frequent engagement in sports betting and had high likelihoods of experiencing gambling problems. Associated psychiatric disorders, especially substance or behavioral addictions, have also been reported. Taken together, the synthesis of findings from this review are an initial step in the direction of identifying individuals at elevated risk for problems associated with sports betting, with further research investigating sports betting and fantasy sports being necessary as the social and technological context of sports betting continues to evolve.

## Data Availability

The data that support the findings of this study are available from the corresponding author upon request.
